# Efficiency and productivity assessment of public hospitals in Greece during the crisis period 2009–2012

**DOI:** 10.1186/s12962-017-0068-5

**Published:** 2017-04-26

**Authors:** P. Xenos, J. Yfantopoulos, M. Nektarios, N. Polyzos, P. Tinios, A. Constantopoulos

**Affiliations:** 1 School of Finance and Statistics, University of Piraeus, 80 Karaoli & Dimitriou Str, 18534 Piraeus, Greece; 2 School of Economics and Political Science, University of Athens, 6 Themistokleous Str., 10678 Athens, Greece; 3 School of Social, Political and Economic Science, University of Thrace, 12 Vasilisis Sofias Str, 67100 Xanthi, Greece

**Keywords:** Efficiency, Hospitals, Productivity, Malmquist, Tobit, Greece, Crisis, C14, D24, H51

## Abstract

**Background:**

This study is an initial effort to examine the dynamics of efficiency and productivity in Greek public hospitals during the first phase of the crisis 2009–2012. Data were collected by the Ministry of Health after several quality controls ensuring comparability and validity of hospital inputs and outputs. Productivity is estimated using the Malmquist Indicator, decomposing the estimated values into efficiency and technological change.

**Methods:**

Hospital efficiency and productivity growth are calculated by bootstrapping the non-parametric Malmquist analysis. The advantage of this method is the estimation efficiency and productivity through the corresponding confidence intervals. Additionally, a Random-effects Tobit model is explored to investigate the impact of contextual factors on the magnitude of efficiency.

**Results:**

Findings reveal substantial variations in hospital productivity over the period from 2009 to 2012. The economic crisis of 2009 had a negative impact in productivity. The average Malmquist Productivity Indicator (MPI) score is 0.72 with unity signifying stable production. Approximately 91% of the hospitals score lower than unity. Substantial increase is observed between 2010 and 2011, as indicated by the average MPI score which fluctuates to 1.52. Moreover, technology change scored more than unity in more than 75% of hospitals. The last period (2011–2012) has shown stabilization in the expansionary process of productivity. The main factors contributing to overall productivity gains are increases in occupancy rates, type and size of the hospital.

**Conclusions:**

This paper attempts to offer insights in efficiency and productivity growth for public hospitals in Greece. The results suggest that the average hospital experienced substantial productivity growth between 2009 and 2012 as indicated by variations in MPI. Almost all of the productivity increase was due to technology change which could be explained by the concurrent managerial and financing healthcare reforms. Hospitals operating under decreasing returns to scale could achieve higher efficiency rates by reducing their capacity. However, certain social objectives should also be considered. Emphasis perhaps should be placed in utilizing and advancing managerial and organizational reforms, so that the benefits of technological improvements will have a continuing positive impact in the future.

**Electronic supplementary material:**

The online version of this article (doi:10.1186/s12962-017-0068-5) contains supplementary material, which is available to authorized users.

## Background

Analysis of efficiency and productivity of the hospital sector has become a considerable concern in Europe. It presents a challenging area of relevant studies because hospitals absorb a large amount of public healthcare spending. In OECD countries and in the European Union of the 27 member states, hospital expenditure represents on the average around 30 and 37% of the total health expenditures in 2012 respectively [1]. The corresponding share in Greece is 47%, indicating a hospital based healthcare system and the need to give greater emphases on operational efficiency and cost containment in order to balance healthcare expenditure at a feasible level [2, 3].

The Greek health system is a combination of an National Health System (NHS) model in the supply-side (consisting of an extensive hospital sector of 138 public hospitals and an underdeveloped primary healthcare sector) and a social insurance system in the demand side (consisting of many health funds that all merged in one, EOPPY (National Organization for the Provision of Healthcare), in 2010. Grants from the public budget finance the ‘fixed’ and contractual expenses (mainly salaries) of public hospitals, while the revenues from the social insurance funds (now consolidate in the single fund EOPPY) finance the variable expenses.

During the crisis period 2009–2012, a reduction of 40% took place in hospital budgets. Additionally, shortages in healthcare workforce and medical supplies have been recorded in the Greek hospital sector [4]. The current efforts of public authorities towards a more efficient allocation of financial and human resources to public hospitals raised questions about the criteria used to evaluate the performance of the Greek healthcare system in the previous years.

Nowadays, Greece has 136 public plus two Non-Governmental Organizations (NGO) hospitals, managed by 85 NHS Trusts, which belong to the Greek National Health System (ESY). During the crisis period 2009–2012, the Greek Ministry of Health attempted to reform public hospitals operations and restrain healthcare expenditure. One such operation was the initiation of department budgets, which offers better expenditure control, more accurate estimation of hospital products and supports productivity enhancement in hospital departments [5]. Furthermore, two major reforms were implemented regarding Greek public hospitals. The first reform was the operational redeployment of the 136 NHS hospitals into 85 Trusts and the second was the implementation of a Diagnosis Related Groups (DRG) prospective reimbursement system, which was introduced in 2011, in order to minimize costs. DRGs are developed in order to identify and price hospital services, based on the diagnosis [6]. Apart from the above measures, additional reforms are in progress in order to restrain healthcare expenditure. For example, the joint purchasing of goods and services by using price–volume agreements can lead to significant decline of healthcare costs. Additionally, the consolidation of NHS hospitals, the adoption of specific policies related to pharmaceuticals and the advancement of public hospitals infrastructure and technology can further contribute to expenditure reductions. At this point, it is important to mention that EOPYY, signs contracts with all Greek hospitals under the KEN-DRG reimbursement system [2, 5].

To the best of our knowledge, the impact of these hospital reforms has not yet been measured. Theoretically, budget cuts are expected to cause a positive shift of efficiency provided that outputs remain stable. Intuitively, since shortages in workforce and medical equipment vary between hospitals, their impact would most probably affect efficiency change rather than technology. The redeployment of hospitals leads to better management of inputs. Therefore, by reducing costs it would most probably increase the overall efficiency of the redeployed hospitals. Moreover, the DRG-based reimbursement system, combined with the pharmaceutical pricing reforms, is expected to create economies of scale which would greatly improve hospital efficiency [7].

### Research objectives

The purpose of this paper is to investigate the dynamics of productivity and efficiency in the Greek Hospital sector over the years from 2009 to 2012. The study is limited in this period, due to the unavailability of more recent data. Moreover, data prior to 2009 were not collected and validated according to international organization principles and guidelines.

We make use of Malmquist Productivity Index (MPI) through data envelopment analysis (DEA) augmented by bootstrapping techniques. The study contributes to the current literature in several possible ways. First, it takes into account all Greek public hospitals (excluding the specialized in psychiatry and pediatrics). Homogeneity is preserved and selection bias is avoided. Second, the data are collected by the Ministry of Health after several quality controls ensuring comparability and validity of the hospital inputs and outputs. Third, our methodology is based on the non-parametric Malmquist productivity analysis developed by Simar and Wilson [8] not previously applied in Greek hospital sector. The great advantage of this method is the estimation of efficiency and productivity change followed by the corresponding confidence intervals. Fourth we decompose the estimated values of productivity into efficiency and technological change components. The above points would provide valuable information to decision makers for effective policy guidance during the crisis period of 2009–2012.

The rest of the paper is arranged in three sections as follows. The first section provides efficiency and productivity measurement concepts, with a brief literature review on healthcare efficiency measurement in Greece and in some other countries. In the following section, the data and the estimated results are presented and discussed. The final section provides the conclusion of the study.

### Hospital efficiency and productivity measurement

The measurement of efficiency and productivity is crucial for hospitals because it allows them to compare the performance of their own organization with that of other hospitals in the same NHS and establish a reciprocal policy of “best practices” in order to improve their own performance [9–13].

Jenu-Appiah et al. [14] and Kirigia and Asbu [15] used two-stage analysis using DEA efficiency measurement and Tobit model in order to examine relationships between hospital inefficiencies and environmental factors. Both studies used cross-sectional data.

Zavras et al. [16], by using DEA, assessed the relative efficiency of 133 primary healthcare services, between 1998 and 1999; the results indicated that the primary healthcare centers that had the appropriate technological capacity to carry out laboratory or radiological examinations had the highest efficiency scores, whereas the medium-sized centers that covered population areas of 10,000–50,000 people performed better than the other primary healthcare units.

In another study, Tsekouras et al. [17], by using Bootstrap DEA, measured the productive efficiency of 39 intensive care units (ICUs) of the Greek Healthcare system for 2004. The purpose of the study was to reveal if new medical technology investment into ICUs had a positive impact; the findings demonstrated that technical efficiency improved but scale efficiency remained unchanged.

Certain studies employ the Malmquist Index methodology and then decompose total factor productivity into technical efficiency and technology change. In Greece, the application of DEA in efficiency and productivity measurement has gained considerable attention by both researchers and policy makers [18, 19]. In a recent study Karagiannis and Velentzas [20] estimated productivity growth for Greek public hospitals for the period 2002–2007 including quality variables in their analysis. They create a quality-adjusted Malmquist productivity index. Their findings indicate reductions both in productivity and quality as well as significant variations between hospitals.

Androutsou et al. [21] measured the performance in seven homogenous specialty clinics across all National Health System hospitals in the Regional Health Authority (RHA) of Thessaly, over the period 2002–2006 with Malmquist Index. Overall productivity progressed in all clinics. Technical change progressed except the general medicine clinics, and diachronically the size of the clinics influences the overall effects on hospital performance. Polyzos [22] analyzed the performance of 117 Greek NHS hospitals by means of DEA, for years 2009–2011. All hospitals, especially middle-sized hospitals showed performance improvements on technical efficiency terms.

This study attempts to make an early assessment of the health reforms in the period 2009–2012 by exploiting the Malmquist methodology which provides a dynamic approach to the assessment of efficiency and productivity of the hospital sector. Additionally, a Random-effects Tobit regression model is explored to investigate the impact of several contextual factors on the magnitude of efficiency in public hospitals.

## Methods

### Data envelopment analysis

Charnes, Cooper and Rhodes (CCR) [23] calculated the efficiency frontier basing their estimates on best practices rather than the average performance in a given sample. Based on their research, Banker et al. [24] introduced the “ Banker, Charnes and Cooper (BCC) model” of efficiency measurement. This model assumes a production technology of variable returns to scale, implying that any proportional change in inputs usage results in variable proportional change in outputs [25]. Specifically, we used the input-oriented approach, since inputs are more easily controlled by hospital administrations, compared to outputs.

According to Simar and Wilson [26], two-stage approach results are inconsistent and biased unless the DEA efficiency scores are corrected by a bootstrapping procedure. Bootstrapping estimates a more robust regression model in order to determine the effect of contextual factors on efficiency [27].

The DEA model can only be applied to multiple DMUs (Decision Making Units: hospitals in our case) on a per-year basis. Therefore, DEA cannot estimate the efficiency change over time. The Malmquist Productivity Index (MPI), which is presented in the next sub-section, overcomes this limitation. In a non-parametric framework the MPI evaluates the efficiency change over time [28].

### The malmquist productivity index

Assuming a list of p inputs and q outputs, the production set is defined in the Euclidean space ${\mathcal{R}}_{ + }^{{{\text{p}} + {\text{q}}}}$ as follows:

1 $$\Xi = \left\{ {\left( {{\text{x}}, {\text{y}}} \right) | {\text{x}} \in {\mathcal{R}}_{ + }^{\text{p}} , {\text{y}} \in {\mathcal{R}}_{ + }^{\text{q}} , \left( {{\text{x}},{\text{y}}} \right) {\text{is feasible}}} \right\}$$

We can define the input requirement set V(y) as the set of all input vectors that can produce the output vector $y \in {\mathcal{R}}_{ + }^{q}$:

2 $${\text{V}}\left( {\text{y}} \right) = \left( {{\text{x}} \in {\mathcal{R}}_{ + }^{\text{p}} |\left( {{\text{x}},{\text{y}}} \right) \in \varXi } \right)$$

Fare et al. [29] determined the input distance function:

3 $${\text{D}}_{\text{i}}^{\text{t}} \left( {{\text{x}}^{\text{t}} , {\text{y}}^{\text{t}} } \right) = { \sup }\{ \uplambda :({\text{x}}^{\text{t}} /\uplambda ,{\text{y}}^{\text{t}} ) \in {\text{S}}^{\text{t}}\}$$

where ${\text{S}}^{\text{t}} = \left\{ {\left( {{\text{x}}^{\text{t}} ,{\text{y}}^{\text{t}} } \right):{\text{x}}^{\text{t}} {\text{can produce y}}^{\text{t}} } \right\}$. Malmquist Total Factor Productivity change index between period t and t+1 as:

4 $$\begin{aligned} &{\text{M}}_{\text{I}} \left( {{\text{X}}^{\text{t}} , {\text{Y}}^{\text{t}} ,{\text{X}}^{{{\text{t}} + 1}} , {\text{Y}}^{{{\text{t}} + 1}} } \right) \\ &\quad = \left[ {{\text{M}}_{\text{I}}^{\text{t}} \left( {{\text{X}}^{{{\text{t}} + 1}} , {\text{Y}}^{{{\text{t}} + 1}} ,{\text{X}}^{\text{t}} , {\text{Y}}^{\text{t}} } \right) \times {\text{M}}_{\text{I}}^{{{\text{t}} + 1}} \left( {{\text{X}}^{{{\text{t}} + 1}} , {\text{Y}}^{{{\text{t}} + 1}} ,{\text{X}}^{\text{t}} , {\text{Y}}^{\text{t}} } \right)} \right]^{1/2} \\ &\quad= \left[ {\frac{{{\text{D}}_{\text{I}}^{\text{t}} \left( {{\text{X}}^{{{\text{t}} + 1}} , {\text{Y}}^{{{\text{t}} + 1}} } \right)}}{{{\text{D}}_{\text{I}}^{\text{t}} \left( {{\text{X}}^{\text{t}} , {\text{Y}}^{\text{t}} } \right)}}\frac{{{\text{D}}_{\text{I}}^{{{\text{t}} + 1}} \left( {{\text{X}}^{{{\text{t}} + 1}} , {\text{Y}}^{{{\text{t}} + 1}} } \right)}}{{{\text{D}}_{\text{I}}^{{{\text{t}} + 1}} \left( {{\text{X}}^{\text{t}} , {\text{Y}}^{\text{t}} } \right)}}} \right]^{1/2}\end{aligned}$$

Equation 4 represents the geometric mean of the two Malmquist indices for periods t and t+1. The first index employs reference technology, which corresponds to period t, while the second index performs the same function, as the first one, for period t+1.

Fare et al. [29] factor the expression (4) into the product of technical efficiency and technological change (frontier shift) as:

5 $$\begin{aligned} &{\text{M}}_{\text{I}} \left( {{\text{X}}^{\text{t}} , {\text{Y}}^{\text{t}} ,{\text{X}}^{{{\text{t}} + 1}} , {\text{Y}}^{{{\text{t}} + 1}} } \right) \\ &\quad= \frac{{{\text{D}}_{\text{I}}^{{{\text{t}} + 1}} \left( {{\text{X}}^{{{\text{t}} + 1}} , {\text{Y}}^{{{\text{t}} + 1}} } \right)}}{{{\text{D}}_{\text{I}}^{\text{t}} \left( {{\text{X}}^{\text{t}} , {\text{Y}}^{\text{t}} } \right)}}\left[ {\frac{{{\text{D}}_{\text{I}}^{\text{t}} \left( {{\text{X}}^{{{\text{t}} + 1}} , {\text{Y}}^{{{\text{t}} + 1}} } \right)}}{{{\text{D}}_{\text{I}}^{{{\text{t}} + 1}} \left( {{\text{X}}^{{{\text{t}} + 1}} , {\text{Y}}^{{{\text{t}} + 1}} } \right)}} \frac{{{\text{D}}_{\text{I}}^{\text{t}} \left( {{\text{X}}^{\text{t}} , {\text{Y}}^{\text{t}} } \right)}}{{{\text{D}}_{\text{I}}^{{{\text{t}} + 1}} \left( {{\text{X}}^{\text{t}} , {\text{Y}}^{\text{t}} } \right)}}} \right]^{1/2} \end{aligned}$$

or

$${\text{M }} = {\text{E}}\; \times \;{\text{T}}$$

where “M” symbolizes Total Factor Productivity Growth index between periods t and t+1, and “E” and “T” represent the technical efficiency change, and the technological change respectively for the same period. Full interpretation of these indices specified to health sector can be found in Jacobs et al. [30] and Adenso-Diaz [31].

By combining each DMUs distance from the efficiency frontier (efficiency change) and the overall shift of the frontier over time (technology change), the Malmquist Productivity Index offers a dynamic approach on handling panel datasets [32].

However, Eq. (4) limits our ability of determining whether changes in productivity, efficiency and technology, really exist or they are merely appearing as such because of the fact that we do not know the actual production frontiers, in which case we must estimate them from the finite sample [26, 33, 34]. For the above reason, a bootstrap estimation procedure for obtaining confidence intervals and correcting the Malmquist Index and its components was employed. The estimation is implemented through the data generating process procedure (DGP), by using a series of pseudo datasets to create a bootstrap estimate. The problems that occur when bootstrapping DEA models are discussed by Simar and Wilson [35]. The bootstrapping procedure concerning Malmquist indices is described in detail at Simar and Wilson [8]. Thus, by obtaining a confidence interval for the Malmquist index and its components it becomes possible to validate whether productivity changes are significant at the desired level of confidence.

However, Simar and Wilson have expressed doubts about the former methodology. They argue that the usual semi-parametric framework is inconsistent in some cases [34]. Using Monte Carlo simulations, they show that since the data generating process cannot be estimated the Tobit regression is inadequate. They propose a truncated regression model and perform single and double bootstrapping, finding that the latter produces better results.

### Regression analysis between inefficiencies and contextual factors

The point of a two-stage analysis of hospital efficiency, is to shed more light on the impact of contextual factors beyond the control of the hospitals on efficiency. Such factors are the operating status of the hospital, the region that is located, etc. In cases where differences across the panel variable have influence on the dependent variable, random-effects models are often used in relevant literature [36–43]. Therefore, in order to explore the potential effect of time as the panel variable, which in this case is expressed in years, we used random- rather than fixed-effects. Besides that, fixed-effects models control for all cannot variables constant across years, such as hospital type, size and RHA, and are therefore unable to measure their effect [44].

The Tobit model ensures lower tail censoring of the distribution that DEA creates. The use of OLS estimation is not appropriate for determining the desired factors of hospital efficiency, because of the nature of the dependent variable (efficiency), which is constrained in the 0–1 interval.

Greene [43] proposed a censoring point at zero for computation purposes and transformed DEA efficiency scores into inefficiency scores left-censored at zero using the equation as follows:

6 $${\text{ineff score}} = \left( {\frac{1}{{{\text{DEA eff}}. {\text{score}}}}} \right)- 1$$

where $${\text{DEA eff}}. {\text{score }} = {\raise0.7ex\hbox{$1$} \!\mathord{\left/ {\vphantom {1 {{\text{D}}_{\text{i}}^{\text{t}} \left( {{\text{x}}^{\text{t}} , {\text{y}}^{\text{t}} } \right)}}}\right.\kern-0pt} \!\lower0.7ex\hbox{${{\text{D}}_{\text{i}}^{\text{t}} \left( {{\text{x}}^{\text{t}} , {\text{y}}^{\text{t}} } \right)}$}}$$.

Consider the linear regression model with panel-data random-effects:

7 $$\begin{aligned} {\text{y}}_{\text{it}}^{ *} & = \upbeta_{\text{i}} {\text{z}}_{\text{it}} + {\text{v}}_{\text{i}} + \upvarepsilon_{\text{it}} \\ {\text{y}}_{\text{it}} & = {\text{y}}_{\text{it}}^{ *} \quad {\text{if y}}_{\text{it}}^{ *} < 0 \\ {\text{y}}_{\text{it}} & = 0 \quad \;\,{\text{if y}}_{\text{it}}^{ *} \le 0 \\ \, & \quad {\text{i}} = \;1, 2, \ldots , {\text{N }} \\ \end{aligned}$$

where i = 1,…,N is the number of DMU’s and t is time, $\upbeta_{\text{i}}$ is the vector of unknown parameters, Z_i_ is the vector of explanatory variables. The random-effects v_i_ are independent and identically distributed (i.i.d.), ${\text{N}}(0, \upsigma_{\text{v}}^{2} )$ and *ɛ*_it_ are i.i.d. ${\text{N}}(0, \upsigma_{\upvarepsilon }^{2} )$ independently of v_i_. The observed data y_it_^*^ represents possibly censored versions of y_it_.

The estimated empirical model is specified in the following equation:

8 $${\text{Tobit}}\left( {\text{ineff}} \right) = \alpha + \upbeta_{\text{i}} {\text{Z}}_{\text{i}} + \cdots + {\text{v}}_{\text{i}} + \upvarepsilon_{\text{i}}$$

where “ineff” is the inefficiency score and ${\text{Z}}_{\text{i}}$ are the following contextual factors: (i) average length of stay (ALS), (ii) bed occupancy rate (OCP), (iii) number of diagnostic procedures (DIAG), (iv) number of patients adjusted by the Roemer index (PAT), (v) type of hospital (1 = Teaching, 0 = Non-Teaching) (TYPE), (vi) three dummy variables concerning hospital size based on the number of beds. Large hospitals are the ones with more than 400 beds (L), medium hospital are the ones containing between 100 and 400 beds (M) and small hospitals are all the rest, having less than 100 beds (S), (vii) seven dummy variables representing each of the seven Regional Health Authorities (RHA) in which Greece is divided (YPE1–YPE7). The RHAs are responsible for planning, coordinating supervising and inspecting all Health Services within the limits of their region. Their aim is to disperse the health sector in order to address problems related to inefficiency in the delivery of healthcare. (viii) four dummy variables signifying the year (YEAR09–YEAR12).

The average length of stay (ALS) is the number of days that an inpatient occupies a bed in the hospital. Positive ALS coefficient would indicate a negative impact on efficiency, since hospital resources remain committed on the same patient. Bed occupancy rate has the opposite impact, because hospitals operate utilizing all available resources. “Diagnostic procedures” include technical and diagnostic procedures, such as blood tests, MRIs, CTs and biochemical exams. If diagnostics are appointed a negative coefficient, it would indicate a positive effect on efficiency. Teaching hospitals are expected to have a positive coefficient, contributing negatively to efficiency. This occurs because healthcare is not their only aim and therefore some resources are spent on the teaching procedure.

### Sampling

On the base of reforms initiated by the memorandum policies, the Ministry of Health has developed a web-based data repository called “ESY-net”. The base includes all Greek hospitals, covering the period 2009–2012 and several variables concerning organizational, medical and financial information. The sample consists of 108 general hospitals for four years (4 years × 108 hospitals = 432 observations). In order to ensure homogeneity of the sample the specialty hospitals (psychiatric, maternity, dermatological and cardiological hospitals) are excluded. ESY-net has been compatible with the international standards of organizations such as World Health Organization, OECD and Eurostat. Grant of access was officially offered to researchers in 2011.

**Fig. 1:**
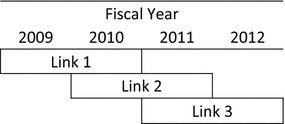
Fiscal years and links

Based on a study by the Centre of Health Economics of the University of York [45], each pair of adjacent years is called “link” throughout the paper. This way, by perceiving consecutive pairs of years as links of a chain, it is easier to explore changes made over time. Links and Fiscal years are shown in Fig. 1.

Given the limitations of the data, the outputs used are: (i) the number of patient discharges adjusted for case-mix with Roemer Index [46]. Roemer et al. provide an adjusted estimate for the average length of stay taking into account the occupancy rate of the hospitals. (ii) the number of diagnostic procedures. The inputs include: (a) the number of doctors, (b) the number of beds, (c) the number of other personnel employed and (d) non-labour expenditures (i.e. pharmaceutical and health technology supplies, etc.) (see Table 1).

**Table 1:** Summary statistics for DEA inputs and outputs for the period 2009–2012

Variables	Definitions	Min.	Max.	Mean	Std. dev.	Coef. var.
Inputs						
Number of doctors	The total number of doctors who are full time employees (FTEs) in the hospitals	8	831	182.98	165.50	0.90
Number of beds	The total number of staffed operational beds in the hospitals	18	949	259.58	218.33	0.84
Expenditure	Total expenditures of hospital other than labor cost (in thousand €)	188	135,740	19769.48	24603.60	1.24
Number of other personnel	The total number of other personnel (non-medical or nurses) who are full time employees in the hospitals	19	1764	275.34	269.26	0.98
Outputs						
Diagnostic procedures	Total number of technical and diagnostic procedures	3940	410,804	97653.74	66012.17	0.68
Number of patients	Total number of patient discharges adjusted for case-mix with Roemer Index	2	114,989	17049.79	18148.73	1.06

All Greek hospitals

Source: Ministry of Health, Greece

The expenditure variable has been deflated by the GDP price deflator (2012 = 100). Following Vassiloglou and Giokas [47], the number of DMUs is greater than three times the number of inputs plus outputs.

### Model specifications

The distance functions that are required in order to obtain Malmquist indices were measured using DEA, assuming constant returns to scale. In order to decompose further the efficiency change into pure efficiency change and scale efficiency change, a variable returns to scale technology (VRS) was considered. Because public hospitals are considered to have smaller ability to control their outputs and more opportunities to lower their inputs, we employed an input-oriented DEA. Moreover, a benchmarking approach was used where the most efficient DMUs were estimated regarding their significance as benchmarks for the inefficient DMUs in the sample data.

## Results

### Productivity

Bootstrapping techniques were implemented in order to eliminate outliers. Supplementary material provided by the authors includes detailed tables containing the MPI, efficiency and technology change scores after bootstrapping (see Additional file 1). Table 2 shows the descriptive statistics of the productivity for 108 hospitals for each time period.

**Table 2:** Descriptive statistics for the Malmquist Productivity Index

	Link 1	Link 2	Link3	Overall
Number of hospitals	108	108	108	108
Min	0.300	0.449	0.520	0.300
Q1	0.585	1.296	0.962	0.795
Q2	0.675	1.490	1.032	1.036
Q3	0.816	1.741	1.151	1.342
Max	1.412	2.874	2.151	2.874
Mean	0.719	1.523	1.078	1.106
Std. dev	0.199	0.387	0.217	0.432

All Greek hospitals, 2009–2012

Source: Ministry of Health, Greece

Analyzing the empirical findings in terms of Malmquist indicator and its components, we witness the following:

In the initial phase of economic crisis in Greece (years 2009–2010) a substantial reduction in the total productivity was observed. Most specifically, the average Malmquist Productivity Indicator (MPI) score is 0.72. Which indicates that the average hospital decreases its production by 28%. Moreover, approximately 91% of the hospitals score lower than unity (stable production) which signifies neutral efficiency.

In the second period, 2010–2011, an impressive hospital productivity gain is observed in the MPI ranging from 0.45 to 2.87, and the average MPI score is 1.52. The second phase represents a period of major healthcare and hospital sector reforms. All Greek hospitals appear to be more productive experiencing productivity gains. Just a small proportion (6%) of hospitals exhibits productivity loss.

Finally, in the third phase 2011–2012 the hospital sector Malmquist values are largely constant—showing neither productivity gains nor losses. Observing productivity trends over the entire period 2009–2012 we witness a productivity improvement of 11% for all hospitals.

**Fig. 2:**
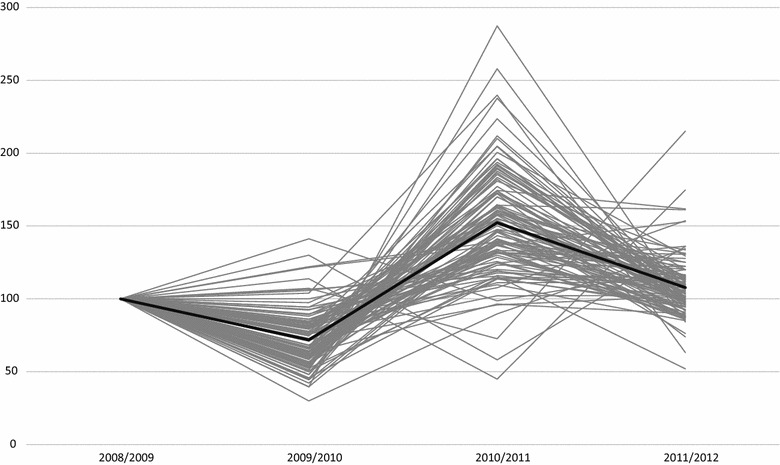
Changes in productivity, 108 Greek public hospitals, years 2009–2012

Figure 2, highlights the path of productivity change over the first Phase of the Greek crisis (2008–2012). The mean values are depicted by a dark thicker line. The productivity in the base year is set to be 100 (MPI = 1). Examining the productivity changes over Link 1 we observe a substantial reduction of 29.1% which is almost homogeneous across the majority of the hospitals since the estimated standard deviation is roughly 0.2.

In Link 2 the launched hospital reforms produced an impressive increase in productivity change of 52.3% However, the productivity gains are not homogeneous across all the hospitals since the majority of the large hospitals present greater values of productivity (higher slopes of productivity lines) in comparison to smaller hospitals. The estimated standard deviation in this period is the highest across the whole path of productivity analysis under consideration (0.387).

During Link 3 a stabilization is observed in the productivity growth followed by a standard deviation of 0.217.

Analyzing the decomposition Malmquist index into Efficiency Change (EC) and Technological Change (TC) we observed in all cases noticeable changes of which the most important is observed in the technological component of the index (see Additional file 1). Tables 3 and 4 exhibit descriptive statistics for the Malmquist Index’s constituent parts for all sample hospitals for all time periods.

**Table 3:** Descriptive statistics for efficiency growth

	Link 1	Link 2	Link3	Overall
Number of hospitals	108	108	108	108
Min	0.485	0.428	0.643	0.428
Q1	0.850	0.966	0.914	0.906
Q2	0.995	1.084	1.000	1.008
Q3	1.143	1.222	1.104	1.168
Max	1.764	1.792	2.349	2.349
Mean	1.007	1.095	1.036	1.046
Std. dev	0.241	0.234	0.218	0.234

All Greek hospitals, 2009–2012

Source: Ministry of Health, Greece

**Table 4:** Descriptive statistics for technology growth

	Link 1	Link 2	Link3	Overall
Number of hospitals	108	108	108	108
Min	0.559	0.992	0.520	0.520
Q1	0.664	1.257	0.991	0.737
Q2	0.705	1.392	1.049	1.045
Q3	0.737	1.523	1.089	1.261
Max	1.066	1.809	1.307	1.809
Mean	0.711	1.388	1.045	1.048
Std. dev	0.076	0.179	0.094	0.304

All Greek hospitals, 2009–2012

Source: Ministry of Health, Greece

Efficiency is related to the combination of input and output resources and the values of Malmquist index indicate a substantial shifting of the hospital production frontier. The empirical findings indicate a slight increase of the overall average efficiency change, and no significant shift is observed in the distribution of the EC for all time periods.

Technological change is the consequence of innovation, that is, the adoption of new technologies by best-practice hospitals. The main finding of this paper, during Link 1, almost every hospital fails to score more than unity in the TC component.

Link 2 exhibits contradictory findings compare to the previous one. The minimum TC score for this period is close to unity signifying the fact that innovation improved in Link 2 for all hospitals, which could indicate that there has been investment in new technologies (methodologies, procedures and techniques) and in the commensurate skills upgrades related to this.

### Quartile persistence over time based on productivity

The following Table 5 depicts how many times hospitals remain in the same quartile. Each hospital can be in the same quartile three times at the most, since three links are considered (2009/10, 2010/11, 2011/12). Quartiles of MPI productivity are represented in the rows and columns represent the number of links. Each number represents how many times hospitals remain in the same quartile. There appears to be small persistence in all quartiles. Persistence in growth would be observable by a large number of hospitals in the last column. No hospitals remain more than twice in the fourth quartile, suggesting that hospitals that rank higher in growth actually do so for a limited time period.

**Table 5:** Quartile persistence of Greek hospitals over time, 2009–2012

	Number of links		
	1	2	3
Lowest growth-Q1	49	16	0
Q2	47	14	2
Q3	35	23	0
Highest growth-Q4	51	15	0

Source: Ministry of Health, Greece

### Probability of transition among quartiles

The probability of shifting between the quartiles of the Productivity Growth is presented in Table 6 for all hospitals. Rows represent the initial quartile and columns the final quartile. Thus, we observe that almost 39% of the hospitals that rank lowest (Q1) in the Productivity Growth measure are among those belonging to Q4 in the following link. Moreover, less than 10% of hospitals initially in Q4 remain in the highest quartile during the following link, indicating that Productivity Growth is indeed not persistent during the time period from 2009 to 2012.

**Table 6:** Quartile transition probabilities (%)

Quartile in link t	Quartile in link t+1			
	Q1	Q2	Q3	Q4
Q1	20.37	18.52	22.22	38.89
Q2	20.37	20.37	27.78	31.48
Q3	25.93	20.37	33.33	20.37
Q4	33.33	38.89	18.52	9.26

Source: Ministry of Health, Greece

### Regression

As mentioned in “Methods”, Tobit regression relates the (in)efficiency scores as the depended variable, both under the assumption of constant returns to scale (CRS) and variable returns to scale (VRS).

Table 7 gives the summary statistics for the independent variables of the model, both nominal and scale, while Table 8 portrays the descriptive statistics for technical efficiency for years 2009–2012.

**Table 7:** Summary statistics for the independent variables of the Tobit Model

Scale variables (N = 108)	Min	Max	Mean	Std. dev.	Coef. var.
Average length of stay (ALS)	1.06	25.63	3.90	1.77	0.46
Bed occupancy rate (OCP)	0.01	1.30	0.61	0.19	0.31

Nominal variables (N = 108)	Frequency
Health prefectures	
Attica	21 (19.4%)
Piraeus & Aegean	17 (15.7%)
Macedonia	14 (13.0%)
Macedonia-Thrace	14 (13.0%)
Thessaly & Central Greece	12 (11.1%)
Peloponnese, Ionian Islands, Epirus and Western Greece	24 (22.2%)
Crete	6 (5.6%)
Hospital type	
Teaching	6 (5.6%)
Non-teaching	102 (94.4%)
Hospital size	
Small (>100 beds)	16 (14.8%)
Medium (100 < beds < 400)	66 (61.1%)
Large (< 400 beds)	26 (24.1%)

All Greek hospitals, 2009–2012

Source: Ministry of Health, Greece

**Table 8:** Summary statistics of technical efficiency (VRS&CRS) by year

Year	Efficiency	Min.	Max.	Mean	Std. dev.
2009	VRS	0.2990	1.0000	0.7337	0.1984
	CRS	0.2473	1.0000	0.6470	0.1904
2010	VRS	0.3256	1.0000	0.7416	0.1879
	CRS	0.3144	1.0000	0.6292	0.1712
2011	VRS	0.3409	1.0000	0.7673	0.1890
	CRS	0.2652	1.0000	0.6731	0.1791
2012	VRS	0.3587	1.0000	0.7568	0.1939
	CRS	0.3319	1.0000	0.6828	0.1759

Bootstrapped. 1000 replications

University hospitals appeared to be less efficient in comparison to non-teaching hospitals which may happened due to the multiplicity of clinical, teaching and research objectives they handle. Similar findings for university hospitals have often being recorded in the literature [48].

Tables 9 and 10 summarize the main findings of two triplets of Tobit models each under a different return to scale assumption.

**Table 9:** Tobit regression results under DEA-CRS assumption

Dependent variable	DEA-CRS efficiency score		
OCP	−2.3468** (0.2073)	−2.6543** (0.25589)	−2.26718** (0.25738)
ALS	0.0156 (0.0275)	0.0076 (0.01378)	−0.0046 (0.0184)
DIAG	<0.000001 (<0.000001)	<0.000001 (<0.000001)	<0.000001 (<0.000001)
PAT	<0.000001 (<0.000001)	<0.000001 (<0.000001)	<0.000001 (<0.000001)
TYPE	0.1452* (0.1355)	0.1766* (0.1523)	0.1833* (0.1734)
L	−1.4192** (−0.1187)	−1.6253** (0.1536)	−1.3237** (0.1735)
M	−0.5155 (0.076)	−0.7264* (0.0562)	−0.4596* (0.0754)
S	(Omitted)	(Omitted)	(Omitted)
YPE1		0.0540 (0.8754)	0.0527 (0.3245)
YPE2		0.4789* (0.3245)	0.8674* (0.7689)
YPE3		−0.3476 (0.2354)	−0.2356 (0.2941)
YPE4		−0.4580 (0.3272)	−0.2134 (0.2873)
YPE5		0.03266 (0.6527)	0.04567 (0.4379)
YPE6		−0.0948 (0.1984)	−0.0456 (0.2197)
YPE7		(Omitted)	(Omitted)
YEAR09			0.2842** (0.0978)
YEAR10			0.3159** (0.0430)
YEAR11			0.0892 (0.0937)
YEAR12			(Omitted)

Standard Error in round brackets

1000 bootstrap replications

*Significance at 10% level

**Significance at 5% level

**Table 10:** Tobit regression results under DEA-VRS assumption

Dependent variable	DEA-VRS efficiency score		
OCP	−1.9836** (0.2459)	−1.9432** (0.2081)	−1.9184** (0.3189)
ALS	−0.004 (−0.0132)	−0.0058 (0.0131)	−0.0189 (0.0137)
DIAG	<0.000001 (<0.000001)	<0.000001 (<0.000001)	<0.000001 (<0.000001)
PAT	<0.000001 (<0.000001)	<0.000001 (<0.000001)	<0.000001 (<0.000001)
TYPE	0.3290* (0.1956)	0.3981* (0.2983)	0.3172* (0.1981)
L	−0.7921* (0.2910)	−0.7234** (0.4289)	−0.7239** (0.1932)
M	−0.2189** (0.0781)	−0.3491** (0.0821)	−0.3280** (0.0729)
S	(Omitted)	(Omitted)	(Omitted)
YPE1		0.0890 (0.1830)	0.091 (0.2379)
YPE2		0.3289 (0.2498)	0.9021 (0.1927)
YPE3		−0.1708 (0.1792)	−0.1739 (0.3902)
YPE4		−0.2893 (0.4920)	−0.4102 (0.3290)
YPE5		−0.0782 (0.1892)	−0.0921 (0.2890)
YPE6		−0.0717 (0.3591)	−0.0792 (0.2198)
YPE7		(Omitted)	(Omitted)
YEAR09			0.0420** (0.0389)
YEAR10			0.0040 (0.0231)
YEAR11			−0.0391 (0.0320)
YEAR12			(Omitted)

Standard Error in round brackets

1000 bootstrap replications

*Significance at 10% level

**Significance at 5% level

There is strong statistical evidence (P > 0.000) that, occupancy rates are a major determinants of hospital (in) efficiency for CRS and VRS assumptions.

Concerning hospital size, high statistical significance is observed (P > 0,0001) in both large and medium categories of the dummy variable. The above statement taking into account the coefficient of medium and large hospitals indicates that small ones appeared to be more ineffective than the rest.

Moreover, no statistical significance has been concerning the RHAs, with the exception of a slight significance at the 5% level for the 2nd RHA as the CRS results indicated. Finally, in both CRS and VRS models there is a statistically significant increase of inefficiency concerning the year 2009.

### Model validation

For the internal (within method) and external (across method) validity of the DEA model the Spearman rank correlation test is implemented, in the same sense as [49]. The DEA methodology was applied using different combinations of inputs and outputs, in order to create two more models apart from the main one (Table 11).

**Table 11:** Models with different combinations of variables

	Inputs				Outputs	
	Doctors	Beds	Oth. personnel	Expenses	Num. of patients	Num. of diag. procedures
1st model	X	X	X	X	X	X
2nd model	X	X	X	X	X	
3rd model		X	X	X	X	X

Source: Ministry of Health, Greece

The Spearman correlation coefficients for the internal validity are high and statistically significant (0.983–0.957). In order to test the external validity of the model, data from different time periods were used (2009, 2010, 2011 and 2012). The results were relatively high and statistically significant (Table 12).

**Table 12:** Model validity test—Spearman rank correlation (Across years)

	2009	2010	2011	2012
2009	–	–	–	–
2010	0.632	–	–	–
2011	0.854*	0.572*	–	–
2012	0.848*	0.665*	0.784*	–

*P value < 0.05

The bootstrap procedure for Malmquist productivity was accessed and implemented through the FEAR package (version 2.0.1) in R [34]. All other computations and results are carried out by the statistical program STATA SE 12.

## Discussion

Several reforms affected production technology. The implementation of departmental budgets developed cost consciousness to the hospital department management. By redeploying the 136 hospitals into 85 Trusts, and by merging the largest Health Insurance Funds into a single purchaser, namely EOPYY, the National Health System exploits economies of scale, regarding both supply and demand. The establishment of the DRG reimbursement system offered the opportunity to improve the pricing procedure of health care services. Moreover, the adoption of specific policies related to pharmaceuticals and the advancement of public hospitals infrastructure and technology contributed further to expenditure reductions. On the other hand, our results indicate that growth lacks persistence across all years and hospitals. This could indicate that the reforms only produced a short-term effect and that long-term efficiency of the NHS is ambiguous.

However, it has not been possible to calculate the effect of each of the above reforms due to lack of data. Such an analysis on future reforms would be an important addition to the current literature. Such reforms could include reducing the capacity of hospitals operating under decreasing returns to scale in order to achieve higher efficiency rates. However, certain social objectives should be considered, such as ensuring adequate access to healthcare facilities, academic and research functions. Emphasis should be placed in increasing managerial and organizational reforms, so that the benefits of technological improvements would create a continuing positive impact in the future.

The reallocation of resources would improve technical efficiency of the system due to cost-containment. Such a reform could include the gradual decrease in the number of doctors followed by subsequent increase in the number of nursing staff.

One of the basic policy makers’ intentions, since the establishment of the Greek NHS in 1983, had been to decentralize its organization and administration, in order to effectively manage the various local healthcare, workforce and financing needs. The Regional Health Authorities were finally established in 2001. Following subsequent legislative amendments, a memorandum law transferred their competences to the regional and local authorities in 2010.

Nonetheless, our results suggest that there is no variation in hospital efficiency among RHAs. This could indicate that RHAs never achieved their initial goal. Their role remained mainly advisory, with little decision-making power, because of their financial and administrative dependence on the central government.

Another policy implication of this study is that, despite the recent cost-containment efforts, certain large public hospitals appear to be leading the way to higher productivity and efficiency. Their “best practice” experience should be identified and adapted by the less productive hospitals, as their example indicates that the economic crisis could provide a window of opportunity for the Greek NHS.

### Limitations

Given the lack of data on quality assessment across the Greek hospitals we strongly recommend the Greek authorities to start collecting information on this important topic. The fact that certain quality aspects of Greek Hospital Organization are not represented in the data set, on which this study is based, might create endogeneity implications.

Another important limitation of this paper concerns availability of data. Even though esy.net has been really beneficent to recent scientific research, our data set has been limited in many ways. More recent data were not yet available to scientists and data prior to 2009 were not collected and validated in accordance with international practices. Moreover, since the establishment of esy.net is fairly recent, it could not provide data concerning DRGs and medical classification of patients, for the period under study.

## Conclusion

This paper attempts to analyze efficiency and productivity growth using bootstrapping of the Malmquist Productivity Indicator (MPI) for public hospitals in Greece. We describe the theory behind MPI and bootstrapping MPI and its decomposition. Having employed confidence intervals, the results suggest that the average hospital experienced substantial productivity growth between 2009 and 2012 as indicated by variations in Malmquist Productivity Indicator. Almost all of the productivity increase was due to technological change and only a minimal part was due to efficiency change.

As far as technological change (innovation) is concerned, it is closely linked to investment. Capital accumulation that influences the adoption of technology by best practice hospitals thereby moves the efficiency frontier. This is confirmed by the fact that there is an average upgrade in technological change for hospitals analyzed that is substantial.

Hospitals operating under decreasing returns to scale could achieve higher efficiency rates by reducing their capacity. However, certain social objectives should be considered, such as ensuring adequate access to healthcare facilities, academic and research functions. Emphasis should be placed in increasing managerial and organizational reforms, so that the benefits of technological improvements would create a continuing positive impact in the future.

## Additional file

**Additional file 1.** Includes detailed tables containing the MPI, efficiency and technology change scores after bootstrapping, flagged for 5% significance.

## Abbreviations

DRG: Diagnosis Related Group
EOPYY: National Organization for the Provision of Healthcare
OECD: Organisation for Economic Co-operation and Development
GDP: gross domestic product
NHS: National Health System
ESY: Greek National Health System
NGO: Non-Governmental Organization
KEN-DRG: Greek Diagnosis Related Group
ESY.net: Greek web-based facility
DEA: data envelopment analysis
SFA: stochastic frontier analysis
CRS: constant return to scale
VRS: variable return to scale
IKA: the Greek Social Insurance Institute
ICUs: intensive care units
BCC: Banker, Charnes and Cooper
ALS: average length of stay
OCP: bed occupancy rate
DIAG: diagnostic and technical examinations
DMU: Decision Making Unit
DGP: data generating process procedure
