# Supplementary material for: Efficiency and productivity assessment of public hospitals in Greece during the crisis period 2009–2012
Source: Cost Eff Resour Alloc. 2017 Apr 26;15:6. doi: 10.1186/s12962-017-0068-5 (PMC5405486; doi:10.1186/s12962-017-0068-5)
Supplement: Supplementary file 1 — Additional file 1. Includes detailed tables containing the MPI, efficiency and technology change scores after bootstrapping, flagged for 5% significance. [file 12962_2017_68_MOESM1_ESM.doc]

| **Table A. Changes in Productivity, 108 Greek public hospitals, years 2009-2012** | | | | | | | |
| --- | --- | --- | --- | --- | --- | --- | --- |
| MPI | | | | | | | |
|  | 2009/2010 | 2010/2011 | 2011/2012 |  | 2009/2010 | 2010/2011 | 2011/2012 |
| DMU #1 | 0.7468* | 1.9372* | 1.2006* | DMU #55 | 0.6637* | 1.5800* | 1.0184 |
| DMU #2 | 0.5846* | 1.6462* | 0.8977* | DMU #56 | 0.5833* | 1.9237* | 1.0725* |
| DMU #3 | 0.9742 | 1.5471* | 1.2506* | DMU #57 | 0.6023* | 1.6090* | 1.0779* |
| DMU #4 | 0.7137* | 1.5846* | 0.9734* | DMU #58 | 0.5398* | 1.7408* | 0.9561* |
| DMU #5 | 0.5614* | 1.6428* | 1.6119* | DMU #59 | 0.6171* | 1.6074* | 1.1990* |
| DMU #6 | 0.5119* | 2.3770* | 1.0075 | DMU #60 | 0.5933* | 1.7718* | 0.9839 |
| DMU #7 | 0.7573* | 1.1460* | 1.1502* | DMU #61 | 0.6057* | 1.5734* | 0.8735* |
| DMU #8 | 0.7269* | 1.3253* | 0.8885* | DMU #62 | 1.3003* | 0.5818* | 1.3598* |
| DMU #9 | 1.001 | 1.2565* | 0.8814* | DMU #63 | 0.8035* | 1.7217* | 0.7390* |
| DMU #10 | 0.7933* | 2.0455* | 0.9158* | DMU #64 | 0.6805* | 1.5821* | 1.3363* |
| DMU #11 | 0.8615* | 1.0968* | 0.8507* | DMU #65 | 0.6186* | 1.7246* | 1.1544* |
| DMU #12 | 0.5026* | 1.1606* | 1.0134 | DMU #66 | 0.5291* | 1.9186* | 1.1455* |
| DMU #13 | 0.7096* | 1.8641* | 1.0372 | DMU #67 | 0.6626* | 1.9630* | 0.9828 |
| DMU #14 | 0.8040* | 1.5393* | 0.9265* | DMU #68 | 0.7110* | 1.4066* | 0.9796* |
| DMU #15 | 0.8295* | 1.0775* | 1.5364* | DMU #69 | 0.8018* | 1.3237* | 1.0801* |
| DMU #16 | 0.7459* | 1.8085* | 1.3061* | DMU #70 | 0.6158* | 1.4339* | 1.1271* |
| DMU #17 | 0.4504* | 1.9138* | 1.0297 | DMU #71 | 0.6364* | 1.3186* | 1.2772* |
| DMU #18 | 0.4500* | 2.1191* | 1.0104 | DMU #72 | 1.1378* | 0.4492* | 1.7475* |
| DMU #19 | 0.6693* | 2.2364* | 0.9906 | DMU #73 | 0.8219* | 1.3974* | 1.1537* |
| DMU #20 | 0.5548* | 1.9006* | 0.9921 | DMU #74 | 0.6210* | 1.6086* | 1.0343* |
| DMU #21 | 0.5433* | 1.7005* | 1.0625 | DMU #75 | 0.9275* | 1.1425* | 0.9456* |
| DMU #22 | 0.4805* | 2.1010* | 0.9758* | DMU #76 | 0.5690* | 1.3687* | 1.5304* |
| DMU #23 | 0.8086* | 1.6230* | 1.0101 | DMU #77 | 0.881 | 1.3210* | 0.9269* |
| DMU #24 | 0.5593* | 1.6461* | 1.1454* | DMU #78 | 0.6029* | 1.8338* | 1.1189* |
| DMU #25 | 0.5333* | 2.0057* | 1.2934* | DMU #79 | 0.7535* | 1.5023* | 0.9223* |
| DMU #26 | 0.9233* | 1.3213* | 1.2013* | DMU #80 | 0.8127* | 1.7443* | 1.6178* |
| DMU #27 | 0.7919* | 1.6366* | 0.9821 | DMU #81 | 0.6636* | 1.2459* | 1.0406* |
| DMU #28 | 0.8489* | 1.3408* | 0.8658* | DMU #82 | 1.0729 | 0.7264* | 2.1512* |
| DMU #29 | 0.6016* | 1.5943* | 1.1323* | DMU #83 | 0.3957* | 2.8739* | 0.9712 |
| DMU #30 | 0.6833* | 1.3227* | 0.8577* | DMU #84 | 0.4810* | 1.8764* | 1.1336* |
| DMU #31 | 0.7708* | 1.1117 | 1.1147* | DMU #85 | 1.2210* | 1.3921* | 0.9959 |
| DMU #32 | 0.6379* | 1.3835* | 1.2514* | DMU #86 | 1.0578* | 1.2023* | 1.1014* |
| DMU #33 | 0.7283* | 1.3899* | 1.1396* | DMU #87 | 0.6651* | 1.2314* | 1.2214* |
| DMU #34 | 0.2996* | 0.8967* | 1.3447* | DMU #88 | 0.8361* | 1.2466* | 0.9078* |
| DMU #35 | 0.7596* | 1.2853* | 0.9623* | DMU #89 | 0.6648* | 1.4618* | 1.016 |
| DMU #36 | 0.4585* | 1.1363* | 0.9642* | DMU #90 | 1.4123* | 0.9867 | 1.2014* |
| DMU #37 | 0.8920* | 1.4768* | 1.3136* | DMU #91 | 0.9436* | 1.1944* | 0.9476* |
| DMU #38 | 0.7682* | 1.6302* | 1.1591* | DMU #92 | 0.5252* | 2.5794* | 0.8416* |
| DMU #39 | 0.8362* | 1.5151* | 1.0498 | DMU #93 | 0.6185* | 1.8533* | 1.0982* |
| DMU #40 | 1.0392 | 2.3983* | 0.6314* | DMU #94 | 0.8605* | 1.2996* | 0.9434* |
| DMU #41 | 0.9441 | 1.5339* | 0.7625* | DMU #95 | 0.7580* | 1.0214 | 1.0224 |
| DMU #42 | 0.6435* | 1.3803* | 0.8738* | DMU #96 | 0.8973 | 1.1772* | 1.1372* |
| DMU #43 | 0.5857* | 1.8915* | 1.0895* | DMU #97 | 0.4997* | 1.6993* | 1.1048* |
| DMU #44 | 0.6554* | 1.8212* | 0.9673 | DMU #98 | 0.8416* | 1.1888* | 1.0743 |
| DMU #45 | 0.6340* | 1.7404* | 0.9589* | DMU #99 | 0.6567* | 1.3726* | 0.9864 |
| DMU #46 | 0.7776* | 1.7288* | 0.9432* | DMU #100 | 0.6270* | 0.9641 | 0.8991* |
| DMU #47 | 0.6379* | 2.0483* | 0.9975 | DMU #101 | 0.5233* | 1.1402* | 1.1182* |
| DMU #48 | 0.4223* | 1.4700* | 0.8866* | DMU #102 | 0.8946 | 1.3318* | 1.3066* |
| DMU #49 | 0.3968* | 1.4575* | 0.8778* | DMU #103 | 1.066 | 1.1557 | 0.5198* |
| DMU #50 | 0.4443* | 1.4738* | 1.0984* | DMU #104 | 0.5511* | 1.4126* | 1.0006 |
| DMU #51 | 0.5633* | 1.9610* | 0.973 | DMU #105 | 0.7171* | 0.9586 | 1.0188 |
| DMU #52 | 0.8141* | 1.5981* | 1.0055 | DMU #106 | 0.7961* | 1.2455* | 1.3612* |
| DMU #53 | 0.6606* | 1.5534* | 1.0895* | DMU #107 | 0.7596* | 1.3126* | 1.1646* |
| DMU #54 | 0.6091* | 1.3944* | 1.2229* | DMU #108 | 1.2151* | 1.3654* | 1.0554* |
| MPI=Malmquist Productivity Index. *Significance at 5% level. 1000 bootstrap replications | | | | | | | |

| **Table B. Changes in Efficiency, 108 Greek public hospitals, years 2009-2012** | | | | | | | |
| --- | --- | --- | --- | --- | --- | --- | --- |
| EC | | | | | | | |
|  | 2009/2010 | 2010/2011 | 2011/2012 |  | 2009/2010 | 2010/2011 | 2011/2012 |
| DMU #1 | 0.9716 | 1.3393* | 1.157 | DMU #55 | 1.0186 | 1.0818 | 0.979 |
| DMU #2 | 0.8110* | 1.2103* | 0.9087 | DMU #56 | 0.8216* | 1.2707* | 1.017 |
| DMU #3 | 1.3026* | 1.1237 | 1.2626* | DMU #57 | 0.9303 | 1.0208 | 1.0677 |
| DMU #4 | 1.0565 | 0.9651 | 0.9105* | DMU #58 | 0.8844 | 1.0401 | 0.9709 |
| DMU #5 | 0.8360* | 1.0508 | 1.5321* | DMU #59 | 0.9457 | 1.1156 | 1.1197* |
| DMU #6 | 0.7557* | 1.5827* | 0.8593 | DMU #60 | 0.8970* | 1.2726* | 0.9526 |
| DMU #7 | 0.9993 | 0.7535* | 1.0075 | DMU #61 | 0.8698 | 1.1765* | 0.8495* |
| DMU #8 | 1.0073 | 0.8987 | 0.8264* | DMU #62 | 1.7640* | 0.4658* | 1.3380* |
| DMU #9 | 1.3253* | 0.8133 | 0.7216* | DMU #63 | 1.2712 | 1.4819* | 0.7622* |
| DMU #10 | 1.084 | 1.2760* | 0.8042* | DMU #64 | 0.9678 | 1.124* | 1.2279* |
| DMU #11 | 1.1672 | 0.7728* | 0.8250* | DMU #65 | 0.8131* | 1.3692* | 1.0484 |
| DMU #12 | 0.6020* | 1.0255 | 0.9375 | DMU #66 | 0.8884 | 1.4001* | 1.1731* |
| DMU #13 | 0.9945 | 1.3760* | 1.1109 | DMU #67 | 0.7853* | 1.5638* | 0.9438 |
| DMU #14 | 1.1405 | 1.1841* | 0.8764* | DMU #68 | 0.9666 | 1.1555 | 0.9163* |
| DMU #15 | 1.1025 | 0.7221* | 1.4145* | DMU #69 | 1.0097 | 1.0361 | 0.9888 |
| DMU #16 | 1.0235 | 1 | 1 | DMU #70 | 0.8751 | 1.0555 | 1.048 |
| DMU #17 | 0.7503* | 1.1057 | 0.9156 | DMU #71 | 0.7260* | 1.0416 | 1.2123* |
| DMU #18 | 0.7048* | 1.2194* | 0.9443 | DMU #72 | 1.2060* | 0.4283* | 1.7258* |
| DMU #19 | 1.0516 | 1.3231* | 0.9539 | DMU #73 | 1.1655 | 1.2276* | 1.0093 |
| DMU #20 | 0.8719* | 1.1334* | 0.9878 | DMU #74 | 0.8753* | 1.2087* | 0.9724 |
| DMU #21 | 0.7734* | 1.2365* | 0.9994 | DMU #75 | 1.3272* | 0.7959* | 0.8705* |
| DMU #22 | 0.6982* | 1.2819* | 0.9487 | DMU #76 | 0.7558* | 0.931 | 1.3754* |
| DMU #23 | 1.1039 | 1.0489 | 0.8428 | DMU #77 | 1.1583* | 1.0562 | 0.905 |
| DMU #24 | 0.7910* | 1.0567 | 0.9968 | DMU #78 | 0.9206 | 1.3287* | 1.0084 |
| DMU #25 | 0.8173* | 1.2941* | 1.1888* | DMU #79 | 1.1821 | 1.0398 | 0.8431* |
| DMU #26 | 1.2770* | 0.8393* | 1.0445 | DMU #80 | 1.1513 | 1.2571* | 1.5672* |
| DMU #27 | 1.0364 | 1.2630* | 1.0172 | DMU #81 | 1.0099 | 0.9821 | 1.0951 |
| DMU #28 | 1.1749 | 1.124 | 0.9418 | DMU #82 | 1.4722* | 0.5376* | 2.3491* |
| DMU #29 | 0.8731 | 1.3405* | 1.2247* | DMU #83 | 0.6650* | 1.7922* | 0.98 |
| DMU #30 | 1.003 | 0.9601 | 0.8130* | DMU #84 | 0.8602 | 1.1938 | 1 |
| DMU #31 | 1.0218 | 0.9239 | 1.1867 | DMU #85 | 1.7187* | 0.9674 | 0.9724 |
| DMU #32 | 0.8844 | 0.9703 | 1.3069* | DMU #86 | 1.2423 | 1.1106 | 0.9584 |
| DMU #33 | 1.0957 | 0.9581 | 1.1672 | DMU #87 | 0.9947 | 0.9705 | 1.1717* |
| DMU #34 | 0.4848* | 0.7432* | 1.1637* | DMU #88 | 1.1295 | 1.2131* | 0.8532* |
| DMU #35 | 1.2148 | 1.1067 | 0.9081 | DMU #89 | 1.0017 | 0.9553 | 0.965 |
| DMU #36 | 0.6601* | 0.9582 | 1.0434 | DMU #90 | 1.6606* | 0.7938* | 1.0469 |
| DMU #37 | 1.2776* | 0.9096 | 1.2550* | DMU #91 | 1.3560* | 0.8675 | 0.9925 |
| DMU #38 | 1.1114 | 0.9891 | 1.0075 | DMU #92 | 0.7723* | 1.7649* | 0.8249* |
| DMU #39 | 1.1151 | 1.0861 | 0.9507 | DMU #93 | 0.8559 | 1.3226* | 1.3142* |
| DMU #40 | 1.4640* | 1.6984* | 0.6430* | DMU #94 | 1.3725* | 0.8169 | 0.9196* |
| DMU #41 | 1.2430* | 1.1484* | 0.6904* | DMU #95 | 0.8369 | 0.8664 | 1.1015 |
| DMU #42 | 0.9122 | 0.9812 | 0.9067* | DMU #96 | 1.0353 | 0.9772 | 1.1209 |
| DMU #43 | 0.9033 | 1.2180* | 1.0796 | DMU #97 | 0.7501* | 1.0697 | 1.03 |
| DMU #44 | 0.9689 | 1.3081* | 0.9605 | DMU #98 | 0.9588 | 1.1353 | 1 |
| DMU #45 | 0.9222 | 1.2193* | 0.9757 | DMU #99 | 0.8981* | 1.092 | 0.9019 |
| DMU #46 | 1.0711 | 1.4351* | 0.9011* | DMU #100 | 1 | 0.9653 | 0.8462* |
| DMU #47 | 0.9581 | 1.3459* | 1.02 | DMU #101 | 0.7698* | 1.1496 | 1.1746* |
| DMU #48 | 0.6770* | 1.0206 | 0.8955* | DMU #102 | 1.2519 | 1 | 1 |
| DMU #49 | 0.5793* | 1.2104* | 0.8353* | DMU #103 | 1 | 1 | 1 |
| DMU #50 | 0.6749* | 0.9656 | 1.0548 | DMU #104 | 0.7554* | 1.0859 | 0.9291 |
| DMU #51 | 0.8439* | 1.2010* | 0.9371 | DMU #105 | 0.9381 | 0.7670* | 1.0855 |
| DMU #52 | 1.2058* | 0.9599 | 0.8684* | DMU #106 | 1.2218 | 0.8400* | 1.3698* |
| DMU #53 | 1.0984 | 1.0647 | 1.0326 | DMU #107 | 1 | 1 | 1 |
|  | 0.8526* | 1.1017 | 1.0458 | DMU #108 | 1.7143* | 1.2596 | 1.1262 |
| EC=Efficiency Change. * Significance at 5% level. 1000 bootstrap replications | | | | | | | |

| **Table C. Changes in Technology. 108 Greek public hospitals. years 2009-2012** | | | | | | | |
| --- | --- | --- | --- | --- | --- | --- | --- |
| TC | | | | | | | |
|  | 2009/2010 | 2010/2011 | 2011/2012 |  | 2009/2010 | 2010/2011 | 2011/2012 |
| DMU #1 | 0.7686* | 1.4465* | 1.0378 | DMU #55 | 0.6516* | 1.4606* | 1.0402 |
| DMU #2 | 0.7209* | 1.3602* | 0.9879 | DMU #56 | 0.7099* | 1.5139* | 1.0546 |
| DMU #3 | 0.7479* | 1.3768* | 0.9905 | DMU #57 | 0.6474* | 1.5762* | 1.0095 |
| DMU #4 | 0.6756* | 1.6419* | 1.0692 | DMU #58 | 0.6103* | 1.6736* | 0.9848 |
| DMU #5 | 0.6715* | 1.5634* | 1.0521 | DMU #59 | 0.6525* | 1.4409* | 1.0708 |
| DMU #6 | 0.6774* | 1.5019* | 1.1725 | DMU #60 | 0.6614* | 1.3923* | 1.0328 |
| DMU #7 | 0.7578* | 1.5209* | 1.1416 | DMU #61 | 0.6964* | 1.3374* | 1.0282 |
| DMU #8 | 0.7216* | 1.4746* | 1.0752 | DMU #62 | 0.7371* | 1.2490* | 1.0162 |
| DMU #9 | 0.7552* | 1.5448* | 1.2214 | DMU #63 | 0.6321* | 1.1618 | 0.9695 |
| DMU #10 | 0.7319* | 1.6031* | 1.1387 | DMU #64 | 0.7031* | 1.4075* | 1.0882 |
| DMU #11 | 0.7381* | 1.4193* | 1.0311 | DMU #65 | 0.7608* | 1.2596* | 1.1011 |
| DMU #12 | 0.8348* | 1.1318* | 1.081 | DMU #66 | 0.5956* | 1.3703* | 0.9765 |
| DMU #13 | 0.7135* | 1.3548* | 0.9336 | DMU #67 | 0.8438* | 1.2553* | 1.0413 |
| DMU #14 | 0.7050* | 1.3000* | 1.0572 | DMU #68 | 0.7356* | 1.2173* | 1.0692 |
| DMU #15 | 0.7524* | 1.4923* | 1.0862 | DMU #69 | 0.7941* | 1.2776* | 1.0923 |
| DMU #16 | 0.7288* | 1.8085* | 1.3061 | DMU #70 | 0.7037* | 1.3585* | 1.0755 |
| DMU #17 | 0.6003* | 1.7308* | 1.1246 | DMU #71 | 0.8766 | 1.2659* | 1.0535 |
| DMU #18 | 0.6384* | 1.7378* | 1.0699 | DMU #72 | 0.9434 | 1.0487 | 1.0126 |
| DMU #19 | 0.6364* | 1.6902* | 1.0385 | DMU #73 | 0.7052* | 1.1383* | 1.143 |
| DMU #20 | 0.6363* | 1.6770* | 1.0043 | DMU #74 | 0.7095* | 1.3309* | 1.0637* |
| DMU #21 | 0.7025* | 1.3753* | 1.0631 | DMU #75 | 0.6989* | 1.4356* | 1.0863 |
| DMU #22 | 0.6881* | 1.6389* | 1.0285 | DMU #76 | 0.7528* | 1.4702* | 1.1127 |
| DMU #23 | 0.7325* | 1.5473* | 1.1985 | DMU #77 | 0.7606* | 1.2507* | 1.0242 |
| DMU #24 | 0.7071* | 1.5578* | 1.1491 | DMU #78 | 0.6549* | 1.3801* | 1.1096* |
| DMU #25 | 0.6526* | 1.5499* | 1.0880* | DMU #79 | 0.6374* | 1.4447* | 1.0939 |
| DMU #26 | 0.7230* | 1.5744* | 1.1501 | DMU #80 | 0.7059* | 1.3875* | 1.0323 |
| DMU #27 | 0.7641* | 1.2958* | 0.9655 | DMU #81 | 0.6571* | 1.2686* | 0.9502 |
| DMU #28 | 0.7226* | 1.1928* | 0.9193 | DMU #82 | 0.7288* | 1.3513 | 0.9157 |
| DMU #29 | 0.6890* | 1.1894* | 0.9245 | DMU #83 | 0.5951* | 1.6035* | 0.991 |
| DMU #30 | 0.6812* | 1.3776* | 1.055 | DMU #84 | 0.5591* | 1.5718* | 1.1336 |
| DMU #31 | 0.7544 | 1.2032* | 0.9394 | DMU #85 | 0.7104* | 1.4391* | 1.0242 |
| DMU #32 | 0.7213* | 1.4258* | 0.9575 | DMU #86 | 0.8515 | 1.0825* | 1.1492 |
| DMU #33 | 0.6646* | 1.4507* | 0.9764 | DMU #87 | 0.6686* | 1.2688* | 1.0424 |
| DMU #34 | 0.6180* | 1.2065 | 1.1556 | DMU #88 | 0.7402* | 1.0276 | 1.064 |
| DMU #35 | 0.6253* | 1.1614 | 1.0598 | DMU #89 | 0.6637* | 1.5302* | 1.0528 |
| DMU #36 | 0.6946* | 1.1858* | 0.9242 | DMU #90 | 0.8505 | 1.2430* | 1.1476 |
| DMU #37 | 0.6982* | 1.6236* | 1.0467 | DMU #91 | 0.6959* | 1.3769* | 0.9547 |
| DMU #38 | 0.6912* | 1.6481* | 1.1504* | DMU #92 | 0.6801* | 1.4615* | 1.0202 |
| DMU #39 | 0.7499* | 1.3950* | 1.1042* | DMU #93 | 0.7227 | 1.4012* | 0.8357 |
| DMU #40 | 0.7099* | 1.4121* | 0.982 | DMU #94 | 0.6270* | 1.5909* | 1.0259 |
| DMU #41 | 0.7595* | 1.3357* | 1.1045 | DMU #95 | 0.9057 | 1.1789 | 0.9282 |
| DMU #42 | 0.7054* | 1.4069* | 0.9637 | DMU #96 | 0.8667 | 1.2047* | 1.0145 |
| DMU #43 | 0.6484* | 1.5530* | 1.0091 | DMU #97 | 0.6662* | 1.5885* | 1.0727 |
| DMU #44 | 0.6765* | 1.3923* | 1.0071 | DMU #98 | 0.8777 | 1.0471 | 1.0743 |
| DMU #45 | 0.6875* | 1.4274* | 0.9828 | DMU #99 | 0.7312* | 1.2569 | 1.0936 |
| DMU #46 | 0.7260* | 1.2047* | 1.0467 | DMU #100 | 0.6270* | 0.9987 | 1.0624 |
| DMU #47 | 0.6658* | 1.5219* | 0.9779 | DMU #101 | 0.6798* | 0.9918 | 0.952 |
| DMU #48 | 0.6238* | 1.4402* | 0.9901 | DMU #102 | 0.7146* | 1.3318* | 1.3066 |
| DMU #49 | 0.6850* | 1.2041* | 1.0508 | DMU #103 | 1.066 | 1.1557 | 0.5198* |
| DMU #50 | 0.6582* | 1.5263* | 1.0414 | DMU #104 | 0.7295* | 1.3008* | 1.0769 |
| DMU #51 | 0.6675* | 1.6329* | 1.0383 | DMU #105 | 0.7644* | 1.2499* | 0.9385 |
| DMU #52 | 0.6752* | 1.6648* | 1.1579* | DMU #106 | 0.6516* | 1.4827* | 0.9937 |
| DMU #53 | 0.6015* | 1.4589* | 1.0551 | DMU #107 | 0.7596 | 1.3126 | 1.1646 |
| DMU #54 | 0.7145* | 1.2657* | 1.1693* | DMU #108 | 0.7088 | 1.084 | 0.9371 |
| TC=Technology Change. *Significance at 5% level. 1000 bootstrap replications | | | | | | | |
